# T cell antigenic recognition sequencing (TAR-seq) enables precise characterization of vaccine-elicited cellular immunity ex vivo and *in vivo*

**DOI:** 10.3389/fimmu.2026.1823042

**Published:** 2026-07-22

**Authors:** Morgan Chaunzwa, GW McElfresh, David Morrow, Gregory J. Boggy, Maanasa Kaza, Sebastian Benjamin, Shana Feltham, Sohita Ojha, Gwendolyn M. Swarbrick, Katherine H. Rott, Roxanne M. Gilbride, Caralyn S. Labriola, Michael K. Axthelm, Vineet Joag, Dylan Kain, Nelita Du Plessis, Gerhard Walzl, Deborah A. Lewinsohn, David M. Lewinsohn, Scott G. Hansen, Louis J. Picker, Benjamin N. Bimber

**Affiliations:** 1 Oregon National Primate Research Center, Oregon Health and Science University, Beaverton, OR, United States; 2 Vaccine and Gene Therapy Institute, Oregon Health and Science University, Beaverton, OR, United States; 3 Division of Infectious Diseases, Department of Pediatrics, Oregon Health & Science University, Portland, OR, United States; 4 Center for Immunity and Immunotherapies, Seattle Children’s Research Institute, Seattle, WA, United States; 5 Department of Pediatrics, University of Washington, Seattle, WA, United States; 6 University Health Network, Department of Medicine, University of Toronto, Toronto, ON, Canada; 7 South African Medical Research Council Centre for Tuberculosis Research; Division of Immunology, Department of Biomedical Sciences, Faculty of Medicine and Health Sciences, Stellenbosch University, Cape Town, South Africa; 8 Division of Pulmonary, Allergy, and Critical Care Medicine, Oregon Health and Sciences University, Portland, OR, United States

**Keywords:** antigen-specific activation, single-cell RNA-seq, T cell activation, T cell receptor (TCR), T cells

## Abstract

**Introduction:**

The functional responses of antigen (Ag) specific T cells are complex, clonotype-specific, context-dependent, and incompletely captured by dominant analytic approaches. Here we present T Cell Antigenic Recognition Sequencing (TAR-seq), a two-part process that uses single-cell RNA + T cell receptor sequencing (scRNA/TCR-seq) to provide a precise and comprehensive characterization of the differentiation state and functional response of T cells specific to a given antigen, *ex vivo* and *in vivo*.

**Methods:**

Many approaches use stimulation and activation-induced markers to identify Ag-specific T cells. We extend this by adding an scRNA/TCR-seq readout to identify the responding TCRs for each subject. We performed rigorous validation using tetramer-sorted T cells and developed a reusable probabilistic model to differentiate TCR-stimulated cells from bystander activation. These TCRs provide molecular barcodes to identify Ag-specific cells from unsorted scRNA/TCR-seq data, irrespective of stimulation, enabling a comprehensive view of their activity.

**Results:**

We applied this method to a cohort of SIV-vaccinated rhesus macaques (RMs). We performed SIV-infected cell recognition assays to identify the SIV-specific TCRs for each RM. We used these TCRs to subset SIV-specific cells and perform precise contrasts. Despite identical vaccination, we identified clone- and subject-level variability in their cytotoxic differentiation and cytokine production after incubation with SIV-infected targets. Per clonotype, the percentage of cells that responded after antigen exposure varied widely. Finally, we used TCRs as barcodes to precisely map the *in vivo* activity of CD8+ T cells 48-120H after SIV challenge. Precise TCR-based identification separated Ag-specific and bystander effects, measuring tissue-specific responses of SIV-specific T cells within 48 hours of infection.

**Discussion:**

Together, our data validate a powerful method to comprehensively understand T cell activity, with relevance to infectious disease, cancer, and autoimmunity, across species.

## Introduction

Cellular immune memory is mediated primarily by the induction and selection of antigen-specific memory T cell populations (1). These cells encode unique T cell receptors (TCRs), where each TCR is capable of specifically recognizing a short peptide presented by a specific MHC molecule, at least for conventional αβ T cells (2–4). After engagement of the TCR to antigen presented on a target cell, the T cell can perform effector functions ranging from direct cytolysis of the target cell to the secretion of cytokines and other factors (5). Inflammation and cytokines can induce non-specific bystander activation of T cells, which is distinct from true antigen-specific recognition (6, 7).

The presence and magnitude of a T cell response do not always correlate with the efficacy of that response. Many factors can influence efficacy, including the antigenic targeting and breadth, affinity to antigen, differentiation state of the T cells, tissue localization and homing, and differential effector properties (8–19). Especially for pathogens where natural protective immunity is rare, such as HIV, malaria, and tuberculosis, considerable research is dedicated to the characterization of protective T cell responses (20–23).

Most techniques to identify and characterize antigen-specific T cells require a tradeoff between specificity and breadth. Major histocompatibility complex (MHC) multimers are soluble fluorescently labeled peptide-MHC (pMHC) complexes that can be used to stain T cells specific to a particular pMHC complex with high specificity (24–26). The weakness of these reagents is that they recognize a single epitope and require non-trivial upfront development to identify the peptide epitope and restricting MHC allele (27, 28). Thus, it is difficult for pMHC multimers to provide comprehensive identification of the T cell response to complex pathogen/antigen, especially across cohorts with diverse MHC genotypes. Further, TCR molecules can be downregulated from the cell surface after stimulation, hampering tetramer binding, tetramers generally require high affinity pMHC/TCR binding, and tetramer binding does not by itself indicate that cell is capable of a functional response (29, 30). Due to these limitations, it is common to use antigenic stimulation and activation-induced markers to identify and characterize antigen-specific T cells. This includes cytokine-capture assays, activation-induced marker (AIM) panels, and assays based on cell proliferation and expansion (31–35). Because stimulation assays do not require upfront knowledge of antigenic peptides, they have greater flexibility than pMHC multimers. The specificity of these surface markers for bona fide TCR-stimulated cells will vary by context, and most marker combinations are expected to include some level of non-specific cells (7, 35).

Single-cell RNA-seq technologies generate full-transcriptome gene expression data with paired TCR sequence. Because scRNA-seq captures the entire transcriptome, these data can measure complex states/phenotypes with higher accuracy than labeling using a handful of surface markers (36). In theory, identifying the set of antigen-specific TCR sequences in an individual would provide stable molecular barcodes capable of specifically identifying T cells with the potential to recognize that antigen, irrespective of the transcriptional state of the cells (e.g., without prior stimulation). Comprehensively identifying these sequences is challenging because TCR sequences are extremely diverse and generally specific to each subject, with limited exceptions for public TCRs (37).

Here we present TAR-seq, a two-step process to comprehensively characterize T cell differentiation and functional properties *ex vivo* and *in vivo*. TAR-seq begins by pairing *ex vivo* antigenic stimulation with a precise scRNA/TCR-seq readout to identify the set of antigen-specific TCR sequences in each subject. We then use these TCR sequences as molecular barcodes for *in silico* identification of T cells with the potential to recognize that antigen, irrespective of prior stimulation. We provide rigorous validation of our methodology, verified using controlled simulation and TCR concordance with tetramer staining. We apply TAR-seq to a cohort of SIV-vaccinated RMs. We identified the SIV-specific TCR repertoire of each RM using physiologically relevant SIV-infected cell recognition assays. We incorporated TCR sequence with gene expression data to demonstrate a wide range of cytotoxic differentiation and cytokine production at the clonotype- and subject-level, despite identical vaccine regimens. Further, we use TCR indexing to precisely quantify the activity of SIV-specific CD8+ T cells 48–120 hours after SIVmac239 challenge, separating true antigen-specific activity from bystander activation. Together, our data validate a powerful method to interrogate *ex vivo* and *in vivo* T cell activity, with relevance to infectious disease, cancer, and autoimmunity.

## Results

### Overview of validation

The first step in TAR-seq is to perform *ex vivo* T cell stimulation assays with a scRNA/TCR-seq readout for the purpose of identifying antigen-specific T cells and their TCR sequences. We demonstrate and validate this in three steps: 1) controlled stimulation and validation of CD8+ T cell activation using tetramers, 2) controlled stimulation of CD4+ T cells, and 3) generation/validation of a reusable probabilistic model to accurately identify bona fide TCR stimulation from scRNA-seq data, using the CD4/8 data as ground-truth.

### Definition of terms

We will use the term ‘Antigen-specific’ (Ag-specific) to refer to T cells that encode a TCR receptor capable of recognizing MHC-bound peptide epitopes from a particular antigen. This study will identify and characterize T cells specific to SIV antigens, CMV antigens, and T cells that respond to superantigen. Antigen specificity is determined by the TCR sequence of the cell. The activation and/or transcriptional state of the cell has nothing to do with Ag-specificity.

The term ‘TCR-stimulated’ will refer to a state induced by antigen-specific stimulation through the TCR. We will define this state based on gene expression changes, and intend this state to be analogous to the antigen-induced activation captured using cytokine production or activation-induced marker assays.

### Identification and validation of gene signatures specific to TCR stimulation in CD8+ T cells

To provide ground-truth data for TCR-associated gene signatures in CD8+ T cells we collected PBMCs from two groups of rhesus macaques (RMs) with distinct characteristics: SIV-vaccinated RMs (n=2) and RMs naturally infected with wild-type rhesus cytomegalovirus (RhCMV) in infancy (n=2). The vaccinated group received a heterologous prime-boost-boost-boost SIV vaccination regimen with four immunizations over a nine-month period (5). We sampled CD8+ T cells four months after the final vaccination, during a period of memory expansion. In contrast, RhCMV is an endemic virus that establishes a lifelong chronic infection in virtually all RMs at a young age. These RMs were 5 and 13 years old at the time of sampling, and thus their RhCMV-specific CD8+ T cell responses have undergone years of maturation due to chronic restimulation. Both SIV-vaccinated RMs express the MHC-I allele Mamu-A1*001 (A*01) and developed responses against the immunodominant SIV Gag_181–189_ CM9 epitope. The RhCMV cohort animals express Mamu-A1*002 (A*02), which presents the immunodominant epitopes RhCMV IE-1_291–299_ VY9 and RhCMV IE-2_313–322_ AN10.

**Figure 1:**
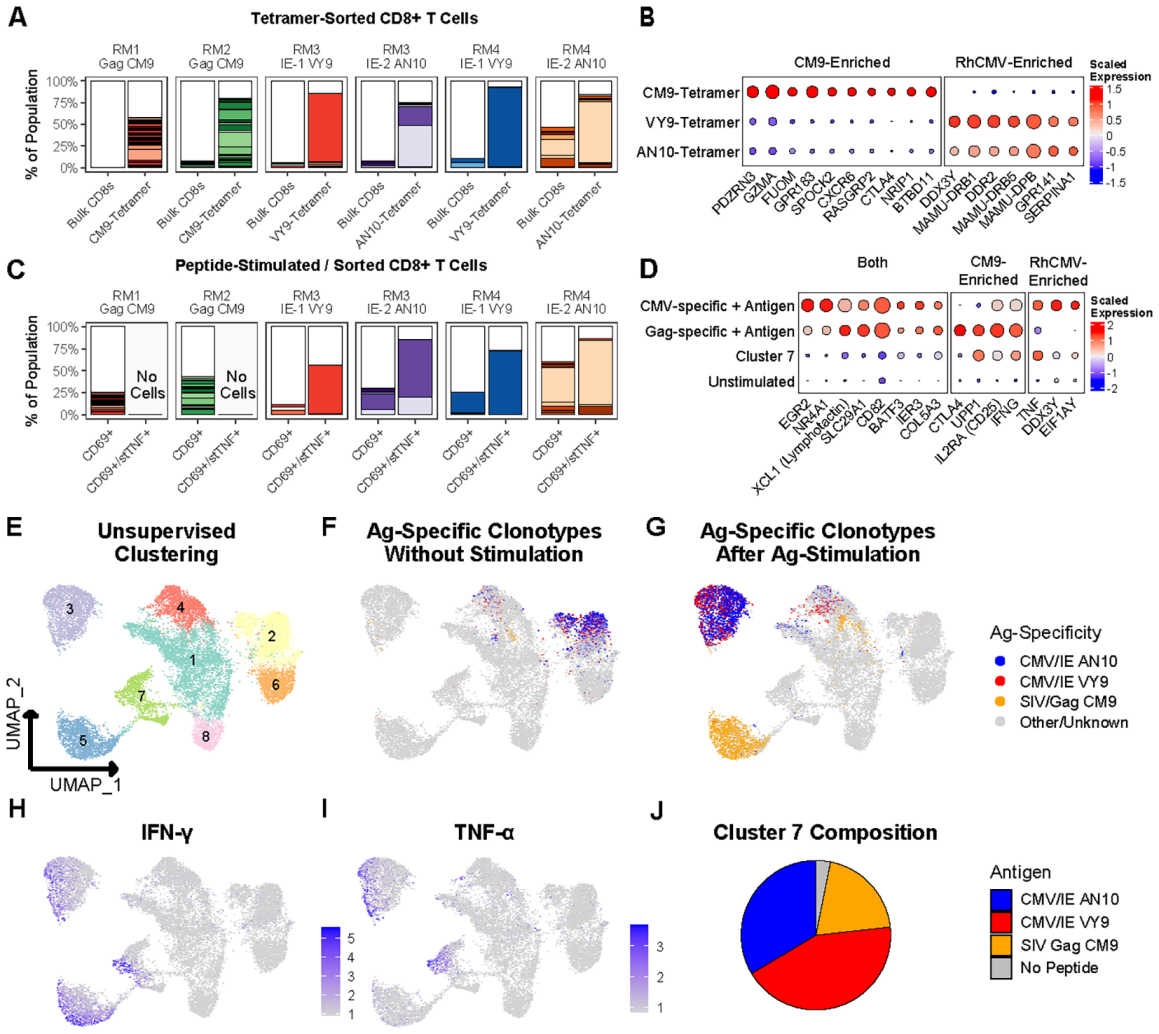
Identification and validation of CD8+ T cell gene signatures specific to TCR stimulation. All panels display single-cell RNA/TCR-seq (scRNA/TCR -seq) data from four RMs, two of which received SIV vaccines. **(A)** The bar plots summarize the TCR clonotypes identified by sorting using the indicated MHC tetramer (right bar). The TCR clonotypes identified from the tetramer-sorted cells were used to quantify the frequency of each clonotype in bulk CD8+ T cells (left bar). **(B)** The dot plot displays the expression of differentially expressed genes between the CM9-, AN10-, and VY9-tetramer sorted CD8+ T cells. Dot size denotes the percentage of cells expressing each gene. **(C)** Cells from each RM were peptide pulsed with the indicated peptide epitope, followed by sorting for either CD69+ CD8+ T cells, or stTNF-α+/CD69+ CD8+ T cells and scRNA/TCR-seq. Cells harboring TCR clonotypes identified in **(A)** are colored using the same color scheme as **(A)**. **(D)** A similar dot plot as **(B)**, summarizing differentially expressed genes from **(C)**. **(E)** The UMAP plot displays dimensionality reduction (PCA/UMAP) from 17,391 CD8+ T cells, including the cells from **(A)** and **(C)**. **(F)** The same UMAP projection as **(E)**, with unstimulated T cells harboring the antigen-specific clonotypes identified in **(A)** are highlighted. **(G)** The same UMAP projection as **(E)**, with peptide-stimulated T cells harboring the antigen-specific clonotypes identified in **(A)** are highlighted. After stimulation with cognate antigen, the antigen-specific clonotypes adopt unique transcriptional states. **(H, I)** The same UMAP projection as **(E)**, with expression of *IFN-γ* **(H)** or *TNF-α* **(I)** highlighted. Upon stimulation with cognate antigen, the antigen-specific T cells localize to clusters enriched for *IFN-γ* and *TNF-α*. **(J)** A pie chart summarizing the composition of cluster 7 grouped by the antigen stimulation, which was enriched for cells expressing *IFN-γ* and *TNF-α* but lacked antigen-specific TCR clonotypes identified by tetramer staining. The enrichment of these clones in peptide-stimulated assays and near absence in control no-peptide assays suggest they represent bystander activation.

We used MHC tetramers to stain PBMCs, followed by sorting and scRNA/TCR-seq of tetramer-positive CD8+ T cells. These data provide both the TCR sequences and the gene expression profiles of these antigen-specific CD8+ T cells at rest (**Figures 1A, B**). Not surprisingly, the frequency of tetramer+ CD8+ T cells varied widely between RMs (range 0.3% to 47%). Of interest, the clonal diversity of the CM9 responses was higher (33–41 distinct clones/response) than the RhCMV responses (6–11 clones/response) and the RhCMV responses each contained 1 or 2 highly expanded clonotypes that represented more than 50% of the response, a feature likely explained by the difference in response maturity (**Figure 1A**). There were also transcriptional differences between the antigen-specific T cells, with higher expression of *Granzyme A* (*GZMA*), *CXCR6* and *CTLA4* in the vaccine-elicited CM9-specific cells, and higher expression of MHC-II genes and the serine protease *SERPINA1* in the RhCMV-elicited T cells (**Figure 1B**). The set of TCR sequences identified by tetramer staining were used to validate bona fide antigen-specific T cells in the subsequent experiments. We use the term ‘antigen-specific’ to refer to cells harboring TCR sequences identified in these tetramer-sorted cells.

We hypothesized that bona fide TCR-stimulated CD8+ T cells undergo significant, predictable transcriptional changes upon stimulation with cognate antigen. SIV Gag_181–189_ CM9 peptide was used to stimulate PBMCs from the SIV-vaccinated cohort, and RhCMV IE-1_291–299_ VY9 and RhCMV IE-2_313–322_ AN10 peptides were used to stimulate the RhCMV-infected RMs (**Figure 1C**). CD8+ T cells were then sorted, followed by scRNA/TCR-seq. We sorted cells using multiple combinations of activation-associated markers, which are expected to have varying degrees of specificity: CD69 (an early but less-specific activation marker), and CD69 with surface-trapped TNF-α (CD69+/stTNF-α), which is highly specific (38, 39). We began by comparing the TCR sequences obtained from each sorted population (**Figure 1C**) against the TCR sequences obtained from the cognate tetramer-sorted data (**Figure 1A**). As expected, the CD69+ populations were enriched for antigen-specific T cells relative to unsorted CD8+ T cells; however, in most cases 75% or more of CD69+ cells were non-specific. In contrast, the CD69+/stTNF-α+ cells were highly specific for antigen, indicated by the overlap with their clonotypes and those identified by tetramer-sorting. While the TNF-trapping assay was successful in both RhCMV-infected RMs, no CD69+/stTNF-α+ cells were detected in either SIV-vaccinated RM.

We performed unsupervised clustering using the gene expression profiles of the 17,931 stimulated and sorted T cells (**Figure 1E**). Cells encoding antigen-specific TCRs, identified from the tetramer-sorted cells, are labeled (**Figure 1F**). After antigen exposure, both CM9-specific and RhCMV-specific T cells formed distinct clusters, indicating they underwent strong transcriptional changes, distinct from resting and bystander T cells (**Figures 1E, G**). The clusters enriched for these cells are also enriched for IFN-γ and TNF-α (**Figures 1H, I**). Of note, the CM9-stimulated, SIV-specific T cells from the SIV-vaccination cohort were primarily located in cluster 5, which expressed IFN-γ, but little TNF-α. That transcriptional phenotype is consistent with the fact that the stTNF-α assay did not yield cells for these RMs (**Figure 1C**). In contrast, the RhCMV-specific TCR clonotypes were primarily located in cluster 3, which was enriched for both IFN-γ and TNF-α. While cluster 7 also expressed IFN-γ and TNF-α, almost none of the T cells in this group encoded TCR clonotypes that matched the tetramer sorted cells. Most cells in this group were derived from peptide-stimulated wells, rather than the control no-peptide wells, suggesting they represent non-specific bystander activation rather than true TCR-mediated antigen-specific activation (**Figure 1J**). We next performed differential gene expression analyses between these groups (**Figure 1D**). This step identified gene programs shared by both antigen-specific, peptide-stimulated clusters, including many established early activation markers such as *CD82*, *NR4A1*, and *IER3* (40, 41*)*. There were also genes enriched in the CM9-specific group, including *CD25* (IL2RA) and *CTLA*, a negative regulator of T cell activation. These markers are largely absent in cluster 7, further supporting the conclusion that these cells represent a bystander or non-specific activation state, which is distinct from the TCR-mediated antigen-specific stimulation observed in clusters 3 and 5. These data provide RNA-centric reference profiles for bona fide antigen-specific activation of CD8+ T cells.

### Identification and validation of gene signatures specific to TCR stimulation in CD4+ T cells

**Figure 2:**
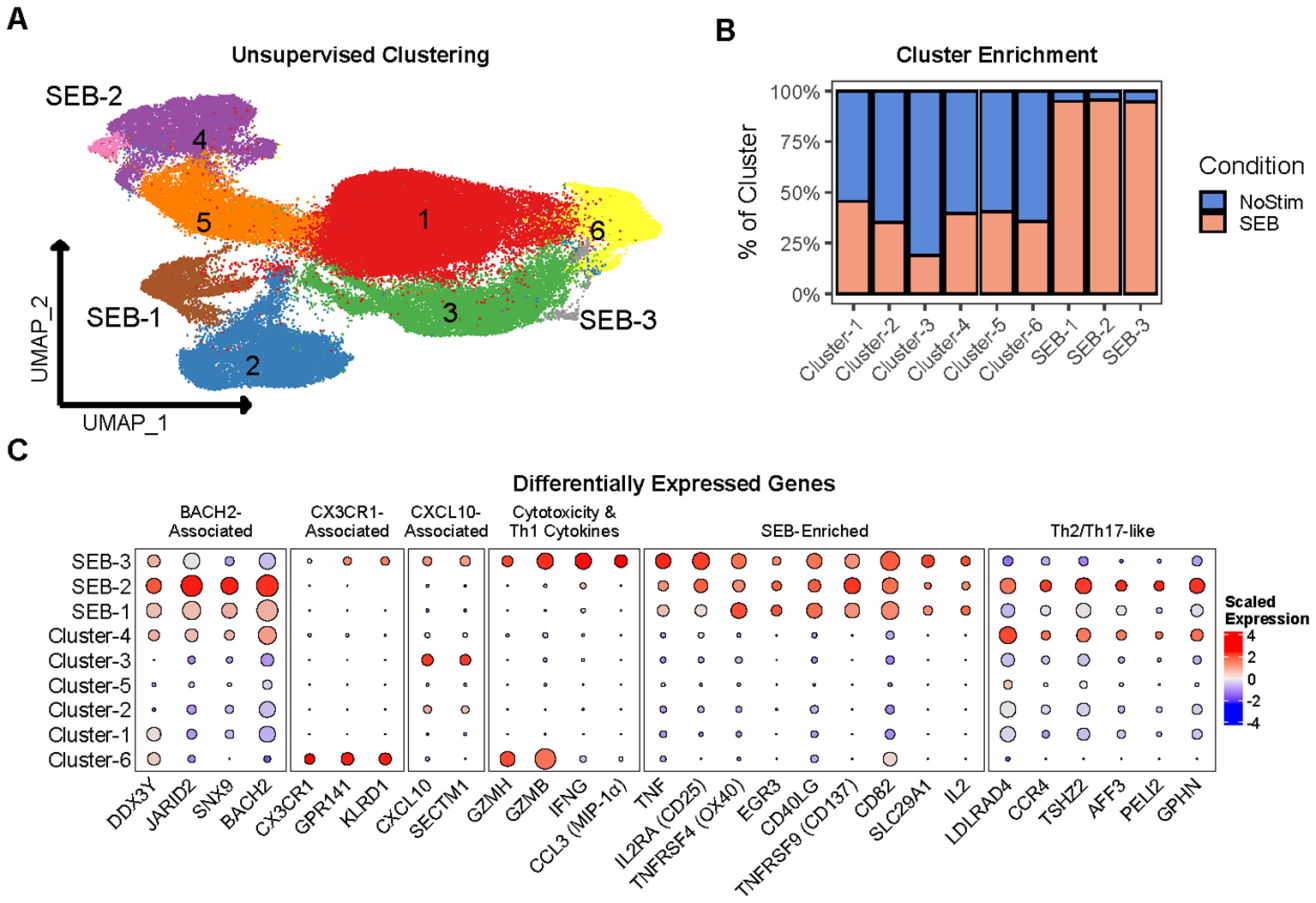
Identification and validation of CD4+ T cell gene signatures specific to TCR stimulation. All panels display single-cell RNA/TCR-seq (scRNA/TCR -seq) data from four RMs, either stimulated with the super-antigen SEB, or unstimulated. These assays were performed in the presence of brefeldin A (BFA). **(A)** The UMAP projection displays dimensionality reduction (PCA/UMAP) for 88,061 CD4+ T cells. **(B)** The bar plot summarizes the proportion of cells for each cluster in **(A)** derived from the SEB or No Stim conditions. Three of the clusters are heavily enriched in SEB-stimulated cells, indicating these transcriptional states are specific to SEB. **(C)** The dot plot summarizes key genes and phenotypic markers with differential expression patterns between the clusters in **(A)**. Dot size denotes the percentage of cells expressing each gene. There is a common gene program upregulated in all SEB-enriched clusters that contains many canonical activation markers, including *IL2*, *TNFRSF4* (OX40), *TNFRSF9* (CD137), *CD40LG*, and *CD82*.

To evaluate the gene programs elicited by TCR-stimulation in CD4+ T cells, we performed an experiment analogous to **Figure 1**. We stimulated T cells from four RMs with staphylococcal enterotoxin B (SEB), a superantigen that binds a subset of TCR V β segments outside of the CDR3 region, thereby eliciting broad TCR-mediated activation (42). Cells were harvested at multiple timepoints: 2H, 4H, 8H, and 24H, followed by scRNA-seq. This experiment was performed in parallel with one series containing brefeldin A (BFA, **Figure 2**) and one without (**Supplementary Figure 1**). Similar results were obtained for each set.

First, we performed unsupervised clustering at multiple resolutions and inspected the data for clusters enriched for SEB-stimulated cells (**Figure 2A**). We identified three distinct clusters enriched for SEB-stimulated cells (SEB-1, SEB-2, and SEB-3), with few unstimulated cells (**Figure 2B**). These clusters contained disproportion frequencies of specific TRB V segments, including TRBV5–1 and TRBV27, consistent with the mechanism of SEB binding and activation (**Supplementary Figure 2**) (42). Each of these clusters contained a mixture of timepoints (**Supplementary Figure 1D**). We then performed differential gene expression analysis between the clusters (**Figure 2C**). All three SEB-enriched clusters specifically expressed established activation markers, including *IL2*, *TNFRSF4* (OX40), *TNFRSF9* (CD137), *CD40LG*, and *CD82 (*35). The most notable characteristic of smaller SEB-2 cluster relative to the others was the expression of chemokine receptor *CCR4*, which has been associated with Th2 and Th17 cells (43). We did not detect significant expression of Th2 or Th17 cytokines in any cells. The third group, SEB-3, was primarily characterized by increased expression of IFN-γ, TNF-α, and cytotoxicity markers Granzymes B and K (*GZMB*, *GZMK*), all consistent with a CD4+ T effector memory (TEM) phenotype. This group was enriched in the 24H timepoint, although it could be detected in all timepoints (**Supplementary Figure 1D**). These data provide reference profiles for TCR stimulation in CD4+ T cells.

### Probabilistic model to identify TCR-stimulated T cells using scRNA-seq data

**Figure 3:**
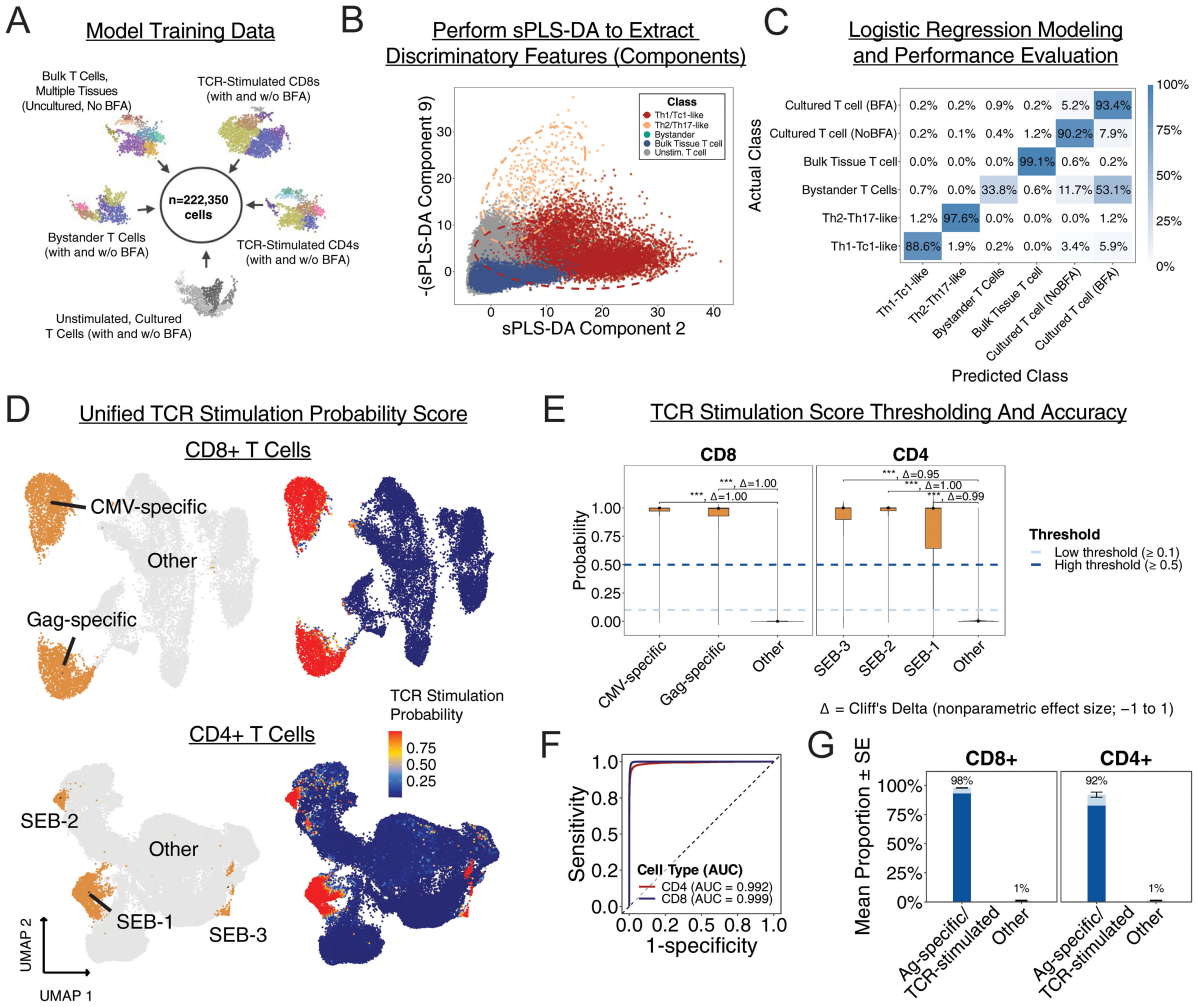
Probabilistic model to identify TCR-stimulated T cells using scRNA-seq data. **(A)** Schematic outlining the sources of training data for the model, which includes validated TCR-stimulated CD4+ and CD8+ T cells, bystander (non-antigen-specific) CD4+ and CD8+ T cells from those assays and cultured/unstimulated T cells. These assays were performed with and without brefeldin A (BFA). Training data also included bulk T cells from tissues. **(B)** A scatter plot illustrating two gene components identified by sPLS-DA, which have discriminatory power to separate TCR-stimulated (e.g. Tc1-Th1-like) from other states. **(C)** A confusion matrix summarizing accuracy of the trained model to assign cells to the indicated classes. **(D)** The CD4+ and CD8+ T cells from **Figures 1**, **2** were scored using the TCR Stimulation Score. The UMAP projections highlight the previously identified antigen-specific/stimulated cells (left, gold), and the TCR Stimulation Score (right). Data are shown for CD8+ T cells (top) and CD4+ T cells (bottom). **(E)** The box plots display the distribution of the TCR Stimulation Scores shown in **(D)**. The dotted lines indicate alternate cutoff thresholds, and *** represents adjusted p-values less than 0.001 **(F)** An AUC plot summarizing the sensitivity and accuracy of the model. **(G)** The bar plots summarize the sensitivity of the model at recovering antigen-specific T cells for each cutoff threshold highlighted in **(E)**. Together, these data validate a probabilistic model that can accurately identify TCR-stimulated CD8+ and CD4+ T cells from scRNA-seq profiles. Panel **(A)** was created in BioRender (44).

To provide accurate and reproducible identification of TCR stimulated cells, we developed and validated a probabilistic scoring framework. The goal was to develop a model-based approach that 1) provides an interpretable, continuous estimate of TCR stimulation probability for each cell, similar to a gating parameter in flow cytometry, 2) identifies the most discriminative gene-level signatures linked to TCR stimulation, and 3) generalizes across major T cell lineages to capture responses in both CD4+ and CD8+ subsets. We began by merging the CD8+ and CD4+ data shown in **Figures 1**, **2** with additional control cells (**Figure 3A**). This included non-specific bystander T cells (with and without BFA), unstimulated cultured T cells (with and without BFA), and bulk uncultured T cells from multiple tissues, comprising cells from 54 RMs (**Supplementary Table 1**). Diverse training data is especially important to avoid conflating stimulation-induced markers with tissue-residency or homing signatures. We grouped the training data into multiple classes, based on transcriptional phenotypes (see methods). Together, this multi-class training dataset provides a structured and biologically grounded foundation for learning TCR-stimulation-specific transcriptional components while controlling experimental and tissue-related confounders.

Next, we applied sparse Partial Least Squares Discriminant Analysis (sPLS-DA), a joint supervised dimensionality reduction and classification method, followed by probabilistic classification (45). After hyperparameter tuning, we identified nine transcriptional components that best separated the curated training classes (**Figure 3B**; **Supplementary Figure 3A**). Each component was defined by a sparse set of highly informative genes, ranging from 20 to 320 genes per component (**Supplementary Table 2**). Together, these captured the principal axes of variation that distinguish TCR-stimulated cells, bystander cells, unstimulated cultured cells, and bulk tissue–derived T cells. Because each component is a weighted gene list, these can be inspected to provide insight into the model. The highly weighted genes in the components include established activation markers, such as *IFNG*, *TNFRSF9* (CD137), *TNFRSF4* (OX40), *CL40LG*, and *CD82* (**Supplementary Figure 3**). We then evaluated whether these components were sufficient to classify cell states by training a multinomial logistic regression model on the nine sPLS-DA variants. The classifier accurately distinguished the training classes and generalized well to a held-out test dataset (**Figure 3C**). Within the stimulated compartment, the model achieved 88.6% accuracy for Th1/Tc1-like cells and 97.6% accuracy for Th2/Th17-like cells (46). Misclassification was mainly between bystander T cells and BFA-treated unstimulated cells. Because both represent non-stimulated classes, this ambiguity does not affect the model’s ability to sensitively and specifically identify TCR-stimulated populations. Across all remaining negative classes, performance exceeded 90% accuracy, indicating robust separation of stimulated versus non-stimulated states across diverse biological and experimental contexts. This model and method have been incorporated into the RIRA R package, via the *PredictTcellActivation* function, to facilitate ease of use by the community (47). Because TCR stimulation induces strong transcriptional effects, it is possible to use simpler methods to identify TCR-stimulated cells, such as scoring cells for discrete gene modules of 6–10 genes using the UCell package (48). The primary advantage of our model relative to simpler approaches is that 1) the model can capture more diversity of activation states, and 2) each gene can be assigned different weights. We performed a comparison between six different gene modules and our model, demonstrating higher sensitivity from our model (**Supplementary Figure 4**).

Having established the classifier’s accuracy and generalizability, we next derived a unified TCR stimulation probability score by summing the model-predicted probabilities across all characterized TCR-stimulation classes (see Methods). When projected onto the original CD4+ and CD8+ datasets, the unified score showed a clear and biologically coherent pattern, with high-probabilities assigned to the TCR-stimulated cells (**Figure 3D**). To quantify the strength of this association, we examined score distributions across all previously characterized stimulated cells (**Figure 3E**). Antigen-specific/TCR-stimulated clusters exhibited markedly elevated scores relative to all other clusters, with Cliff’s delta values ≥ 0.95 for each cluster when contrasted against the remainder of the dataset, indicating a dominant and consistent separation between stimulated and non-stimulated populations. Complementing these distributional analyses, ROC curves stratified by lineage demonstrated AUC values > 0.99 for both CD4+ and CD8+ T cells (**Figure 3F**), further demonstrating the high discriminative power of the unified score in distinguishing antigen-responsive states.

Building on this specificity, we next evaluated thresholds suitable for gating. Using cluster-level score distributions and effect sizes, we evaluated two thresholds (0.5 and 0.1), which provide trade-offs between sensitivity and specificity (**Figure 3G**). Even at the more permissive threshold of 0.1, the unified score recovered >90% of antigen-specific cells for both CD4+ and CD8+ lineages (with slightly better performance for CD8+ T cells), while yielding only ~1% background (**Figure 3G**). These results demonstrate that the unified score supports principled, flow cytometry–like gating strategies with tunable stringency depending on analytical goals and can be used for identifying TCR-stimulated cells.

The TCR activation model is trained to identify transcriptional signatures elicited rapidly after TCR stimulation (~2-8H). This provides high specificity in this controlled setting, but the expression of many of the genes used in the model wanes between 8 and 24H after stimulation, especially cytokine genes (**Supplementary Figure 3B**).

### Application of TAR-seq probabilistic model to human data

RMs and humans are closely related species, with strong conservation in T cell biology. To demonstrate the applicability of the TAR-seq model to human T cells, we isolated T cells from three donors with prior *Mycobacterium tuberculosis* (*Mtb*) exposure and incubated them with autologous dendritic cells (DCs) that were either *Mtb-*infected or mock-infected. CD3+ T cells were sorted and scored using our model (**Supplementary Figure 5**). These data show strong overlap between IFN-γ and TNF-α production and cells scored as TCR-stimulated by the model. This outcome is not surprising given the high degree of conservation across species; however, it demonstrates that this approach can be applied to both human and RM data.

### Application of TAR-seq to SIV vaccination and challenge

**Figure 4:**
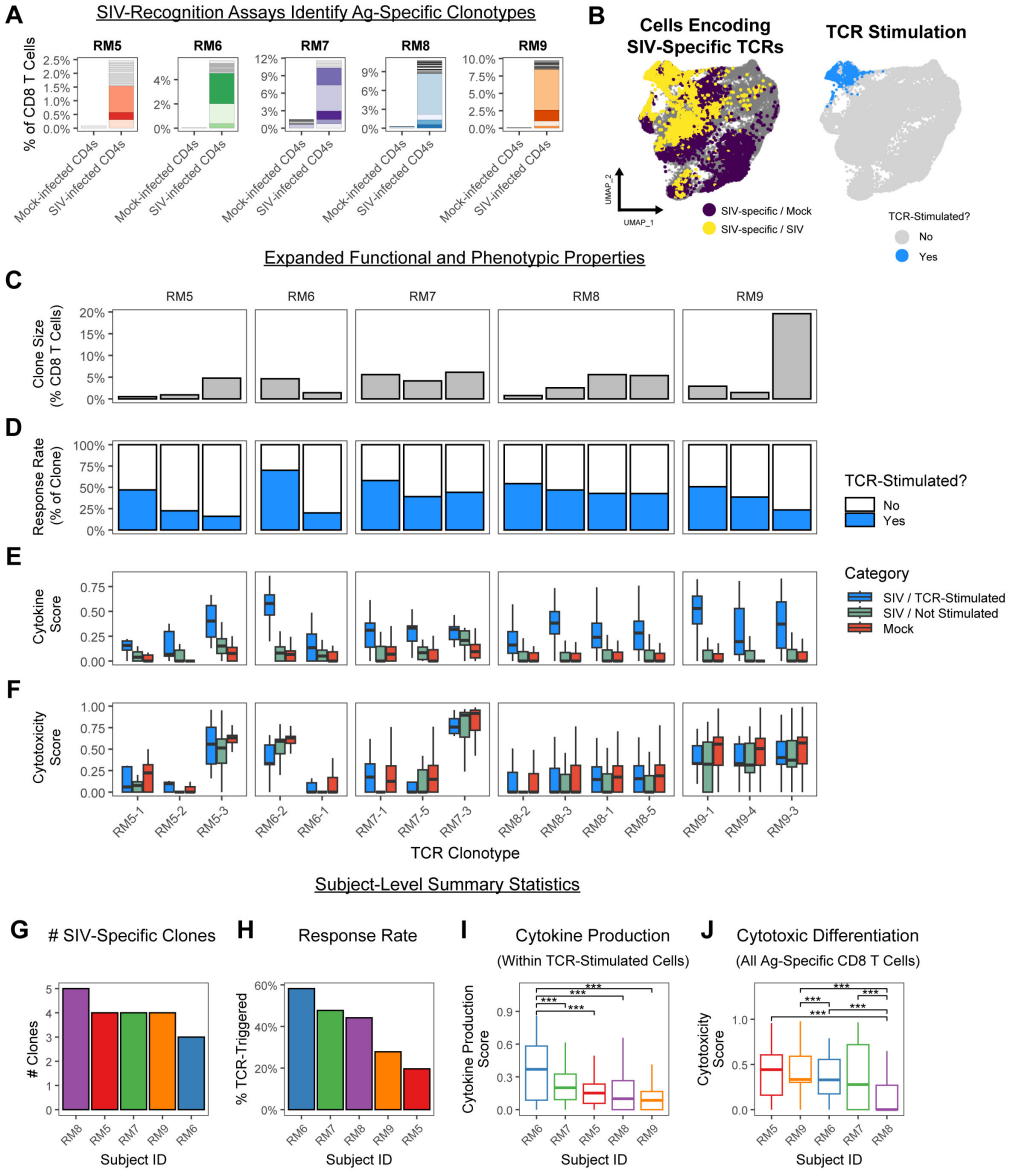
Comprehensive identification and characterization of SIV-specific clonotypes using infected cell recognition assays. All panels display scRNA/TCR-seq data from five RMs that were vaccinated with RhCMV/SIV vectors. Isolated CD8+ T cells were incubated with either SIV-infected autologous CD4+ T cells or mock-infected CD4+ T cells, followed by scRNA/TCR-seq. TCR-stimulated cells were identified using the probabilistic model described in **Figure 3**. **(A)** The plots display the identify and frequency of clonotypes stimulated by each assay. This identified the SIV-specific clonotypes for each RM. **(B)** The left-hand UMAP plots display dimensionality reduction (PCA/UMAP) using all CD8+ T cells from both SIV-infected and Mock-infected CD4s. Cells encoding an SIV-specific TCR, defined by **(A)**, are highlighted, regardless of the activation state of that cell. The right-hand plot is an identical UMAP projection, with each cell colored based on whether that cell scored as TCR-stimulated. **(C)** The plots display the total frequency of each SIV-specific clonotype (including cells that responded to SIV-infected cells and those that did not), as a percentage of total CD8+ T cells. **(D)** The plots display the fraction of cells within each clonotype that responded to SIV-infected CD4+ T cells. **(E)** Cells were scored for a cytokine gene module (*TNF-α*, *IFN-γ*, *MIP-1α*, *MIP-1β*, *NFKBID*, and *TNFRSF14*). The boxplots summarize cytokine production for each clone, grouped by stimulation and antigen response. **(F)** Cells were scored for cytotoxicity using granzyme-based module. The boxplots display analogous data to **(E)** for this score. **(G)** The bar plot summarizes the number of distinct SIV-specific CD8+ T cell clones per RM. **(H)** The bar plot summarizes the response-rate data from **(D)** per RM. **(I)** The box plots summarize the cytokine production data from **(F)** per RM. The data are limited to just clones that responded to SIV-infected cells. **(J)** The bar plot summarizes the cytotoxic differentiation data from **(F)** per RM. The *** in I and J represent adjusted p-values less than 0.001. Collectively, these data illustrate the usage of SIV-infected cell recognition assays with a scRNA/TCR-seq readout to characterize the SIV-specific T cell repertoire and functional properties of these cells.

Our experiments thus far employed simplified systems to validate a framework to accurately identify antigen-specific T cells and their TCRs after antigen-exposure *ex vivo*. To demonstrate the power of this approach in a more diverse situations, we generated a cohort of five RMs vaccinated with rhesus cytomegalovirus SIV (RhCMV/SIV) vectors. We isolated CD8+ T cells from each RM and incubated them with autologous SIV-infected CD4+ T cells, followed by scRNA/TCR-seq. The SIV-infected CD4+ T cells provide a more complete set of antigenic targets and are a more physiologically relevant stimulus than peptide-pulsed cells. After stimulation, CD8+ T cells were scored for TCR activation using the probabilistic model, identifying 4–7 dominant SIV-specific CD8+ T cell clones per RM (**Figure 4A**). We acknowledge that these assays will only detect: 1) clonotypes present in the sample, and 2) clones that are activated in response to antigen. It is possible that additional SIV-specific clonotypes are restricted to tissues or have low frequency in PBMCs at the timepoint we sampled. It would be possible to perform SIV recognition assays using samples from multiple timepoints, or using cells isolated from other tissues.

A key difference between scRNA/TCR-seq and flow cytometric methods is that once the TCR sequences are identified, these TCRs can be used to identify the SIV-specific T cells, including those that did not respond to stimulation (**Figure 4B**). The combination of TCR and gene expression data can be used to contrast functional properties between clonotypes (**Figures 4C–F**). The clonal size differed widely between clonotypes (**Figure 4C**). Interestingly, the proportion of cells per clonotype that responded to SIV-infected CD4s also varied considerably by clonotype, from 16.0% to 88.2% (**Figure 4D**). This property, which compares cells with the potential to recognize an antigen against cells that mount a response to antigen, is extremely difficult to measure using most techniques. These data can be used to study not only how T cells respond, but why they fail to respond. As demonstrated in **Figures 1**, **2**, our T cell activation model identifies TCR stimulation using a broader gene set than just measurement of cytokine production. Nonetheless, cytokine production is an important functional measure that can be contrasted against TCR stimulation. While all CD8+ T cell clonotypes in this cohort produced cytokines upon TCR stimulation, not all clones performed equally (**Figure 4E**). Clone RM5–1 had almost no elevation in cytokine production relative to unstimulated cells, which could impact antiviral efficacy relative to other T cell clonotypes. Cytotoxic differentiation, measured using a granzyme-based gene module, also varied across the clonotypes, and could indicate differential ability to lyse virally infected cells (**Figure 4F**). Interestingly, while the functional properties of the SIV-specific CD8+ T cell clonotypes were uniform in some RMs (RM8, RM9), in others (RM5, RM6, and RM7), we observed a wide range in cytotoxic differentiation between clonotypes.

While **Figures 4C–F** illustrates functional properties at the clonotype level, these can be summarized to the subject/animal level (**Figures 4G–J**). Even in this small proof-of-concept cohort, we identified statistically significant differences between RMs in terms of cytokine production (**Figure 4I**) and cytotoxic differentiation (**Figure 4J**). While the small sample size prevents overly broad conclusions from this dataset, this illustrates how the pairing of TCR and gene expression data can be used to quantify functionally relevant gene programs. This high-resolution characterization of vaccine elicited T cells could be applied to larger vaccine studies, creating precise immune measurements that could be correlated with disease outcome and lead to a more nuanced understanding of T cell mediated protection.

### TAR-seq can precisely characterize SIV-specific T cell function *in vivo*

Next, we applied TAR-seq to characterize activity of SIV-specific T cells *in vivo*, after SIV challenge. The five SIV-vaccinated RMs were challenged intravenously with SIVmac239M, followed by necropsy at 48–120 hours-post-infection (HPI). These represent extremely early timepoints, during which T cells first encounter virally infected targets. Plasma viral loads ranged from 10^3 to 10^6 copies/mL, strongly correlated with the duration of infection. CD8+ T cells were collected from multiple tissues at necropsy, as well as pre-challenge PBMCs, followed by scRNA/TCR-seq of bulk CD8 T cells. SIV-specific T cells were identified by matching their TCR sequences to the clonotypes identified *ex vivo* (the logic used to map between the TCR library and *in vivo* data is described in the methods section). Unlike controlled *ex vivo* stimulations, the CD8+ T cells isolate here were exposed to variable levels of antigen (if at all), with unpredictable kinetics and thus are expected to have varied gene expression profiles.

**Figure 5:**
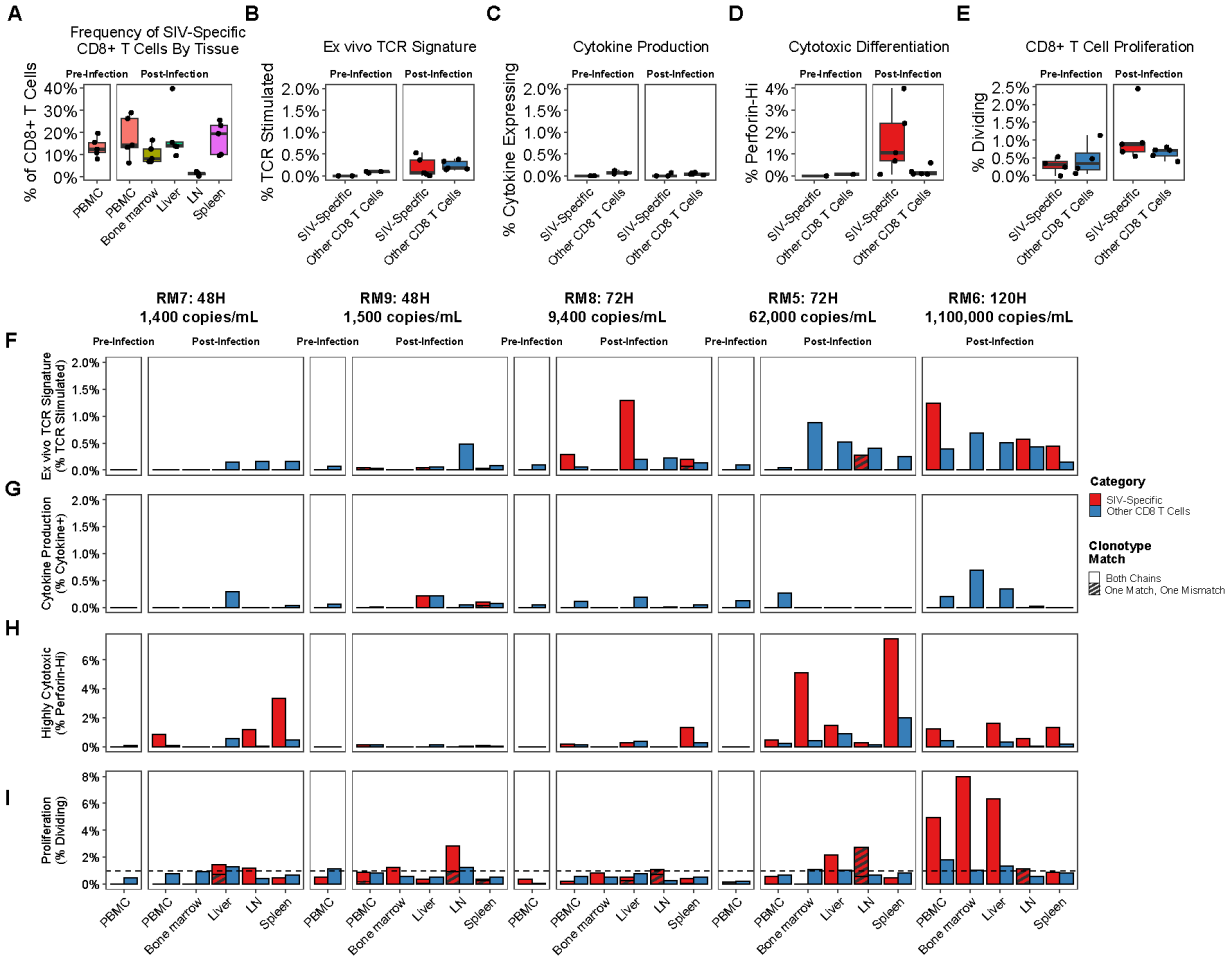
Precise characterization of SIV-specific T cell activity *in vivo*. All panels summarize CD8+ T cell scRNA-seq data from SIV-infected RMs. In each RM, cells are classified as SIV-specific if their TCR sequences match clonotypes identified from *ex vivo* SIV-recognition assays in **Figure 4**. **(A)** The box plots summarize the frequency of T cells encoding the SIV-specific TCRs across each tissue, including pre- and post-infection. **(B–E)** Each panel summarizes CD8+ T cells positive for the indicated signature of activation. **(F–I)** Each panel displays similar data as **(B–E)**, split by RM and tissue. Each RM was necropsied at the indicated post-infection timepoint (48–120 HPI), with the indicated PVL. While most cells were classified as SIV-specific using alpha and beta chains, we identified a small subset of cells that shared one of two chains with a known SIV-specific clone **(F–I)**, diagonal background). Each row represents a distinct measurement of T cell activation, analogous to **(B–E)**. These data illustrate the ability to precisely measure relevant functional properties of antigen-specific T cells *in vivo* and to separate their activity from signatures elicited in bystander cells.

The ability to precisely identify SIV-specific T cells using TCR sequence, instead of relying on activation markers, allows the identification of Ag-specific T cells regardless of their activation state and can more cleanly separate Ag-specific and bystander activation. The frequency of SIV-specific cells was highly variable between tissues, with the highest enrichment in liver and spleen and wide variation in frequencies between RMs (**Figure 5A**). Next, we scored cells using a suite of other gene expression programs associated with T cell activation and effector functions (**Figures 5B–E**). To establish basal levels of these gene programs in healthy RMs, we also scored a previously published control dataset (**Supplementary Figure 6**) (47). When cells were scored using our early TCR-stimulation model, the rates were low in all samples, suggesting that either most cells have not encountered antigen, or they encountered it more than 8 hours prior to sampling (**Figures 5B**, **F**). Likewise, T cells with expression of the cytokine gene module were extremely rare (**Figures 5C**, **G**). Our *ex vivo* data showed rapid downregulation of cytokine genes within hours of the initial TCR stimulation (**Supplementary Figure 3B**), and thus cytokine RNA expression may not be a robust marker of antigenic stimulation *in vivo*. While we could not detect a change in cytokine production, the level of cytotoxicity (measured by perforin-hi cells) increased after SIV-infection relative to pre-challenge, and this increase occurred in SIV-specific CD8+ T cells (**Figures 5D**, **H**). The level of change varied between RMs. RM7 showed increased cytotoxicity of spleen and lymph node SIV-specific CD8+ T cells at 48 HPI, while RM9 (which had comparable viral loads at 48 HPI) had none. Both spleen and bone marrow of RM5 (72 HPI) showed a strong increase in perforin that was limited to SIV-specific T cells. Cell proliferation is also used as an indication of T cell activation, although it is not necessarily driven by true antigen-specific recognition (49). There was an overall increase in proliferation for both SIV-specific and other CD8+ T cells after SIV challenge, which is not surprising (**Figure 5E**). However, inspection of individual samples revealed a strong increase in proliferation within SIV-specific T cells of RM6 (120 HPI), a feature that would not be apparent without TCR-resolution data (**Figure 5I**). RM6 represents the latest timepoint in this cohort, and this is consistent with proliferation being induced later, downstream of initial antigenic exposure.

Because αβ T cells express two independent chains, it is possible to categorize cells as SIV-specific by matching one or both chains. Most cells in this dataset were matched using both chains (**Figures 5F–J**, solid background). Of interest, we identified increased proliferation, particularly in lymph nodes, of CD8+ T cells encoding one chain that matched the previously identified SIV clonotypes and one that was distinct (**Figure 5I**, diagonal background). It is tempting to speculate that this reflects the expansion of rare memory populations; however, this cannot be verified with our current dataset.

These data demonstrate that personalized TCR libraries can be combined with scRNA/TCR-seq to precisely measure the functional state of antigen-specific T cells extremely early after infection and to separate the activity of these cells from non-specific bystander T cells. These precise measurements of vaccine-elicited T cell activity may identify characteristics that differentiate protective from non-protective T cell immunity.

## Discussion

T cells are important immune effectors and one of the main mediators of vaccine-elicited immunity. For many infectious diseases, including HIV, tuberculosis, and malaria, simply eliciting a T cell response is not sufficient to confer protection (8–19). Significant resources are dedicated in these fields toward understanding what constitutes an efficacious T cell response to a given pathogen, which requires a more nuanced understanding and measurement of T cell activity in that context (20–23). Many of the dominant approaches to study T cell activity are indirect, relying on activation induced states (e.g., proliferation, MHC-II expression, or activation-induced surface markers) to identify and quantify T cell engagement (31–35). There is almost always a basal level of T cells expressing these markers *in vivo*, and expression of many markers can be triggered through both bystander signaling and bona fide antigen recognition (6, 50). Precise separation of true antigen-specific from basal or non-specific activation is essential when the magnitude of the antigen-specific response is low, such as early timepoints after pathogen exposure.

Here we present T Cell Antigenic Recognition Sequencing (TAR-seq), an alternative paradigm. TAR-seq has two steps. First, we stimulate T cells from immunized subjects with antigen *ex vivo*, followed by scRNA/TCR-seq. Analogous to many T cell assays, we used activation-induced gene expression changes to identify T cells specific to the antigen, which provided the set of pathogen-specific TCRs in each subject. We developed a reusable probabilistic model that identifies changes elicited rapidly (2-8H) after TCR stimulation, providing high specificity. The second step of TAR-seq acts in reverse. Rather than relying on activation-induced markers, we performed scRNA/TCR-seq on bulk T cells and matched their TCRs against the antigen-specific TCRs identified *ex vivo*. This robustly identified SIV-specific T cells, irrespective of activation state, allowing precise evaluation of vaccine-elicited T cell activity *in vivo*.

We demonstrated the power of this approach using a cohort of vaccinated, SIV-infected RMs. We performed *ex vivo* stimulation using autologous SIV-infected CD4 T cells, providing a comprehensive identification of SIV-specific TCR clonotypes in each RM. The gene expression profiles of these cells provided detailed functional information, revealing subject- and clone-level differences in cytokine production and baseline cytotoxic differentiation, despite receiving an identical vaccine regimen. These measurements could be combined with outcome data to better understand characteristics of a protective CD8 T cell response to HIV/SIV. While we demonstrated this approach using RM and SIV, it could be applied to other species, and to study T cell responses to pathogens, autoimmunity, or cancer.

The success of a vaccine ultimately depends on the activity of immune cells *in vivo*. TCR indexing allows an extremely precise window into CD8+ T cell activity in the days following SIV challenge, and the ability to contrast SIV-specific and bystander cells. We identified increases in cytotoxic differentiation and proliferation that were limited to SIV-specific T cells, with differences between subjects. This level of resolution can be used to address many difficult and important questions. TCR and molecular readouts can contrast the level of T cell engagement between tissues, which could be contrasted against viral measurements. For vaccine development, contrasting these precise T cell phenotypes with outcome can identify correlates of protection.

There are several limitations to this study. TAR-seq relies on the set of antigen-specific TCRs identified using controlled *ex vivo* stimulation. This study identified antigen-specific T cells using SIV-infected cell assays with CD8+ T cells derived from blood. There are many factors that could result in imperfect or potentially biased detection of clonotypes. The Ag-specific clonotype must be present in the sample used for scRNA/TCR-seq, which is further restricted by the number of cells that can be sequenced using droplet-based scRNA-seq methods like 10x Genomics. Clonotypes that are either extremely rare or restricted to specific tissues will not be detected. Alternate approaches that can obtain TCR sequences from far larger numbers of T cells partially mitigate this issue; however, these approaches generally do not acquire paired gene expression and TCR sequence and thus rely on lower fidelity readouts such as clonal expansion to infer antigen-specificity (51). For either of these approaches, it may be advantageous to perform cell sorting after Ag-stimulation to enrich for activated cells, although this carries the potential risk of missing or biasing against clones that do not up-regulate the markers used for enrichment. Using any approach, the TCR library may not represent every possible clonotype; however, it does not need to be complete to provide a powerful tool. A key feature of TAR-seq is that it generates paired gene expression and TCR sequence data, which can be used to interpret unexpected results. If T cell clonotypes not present in the TCR library exhibit signatures of activation *in vivo*, their Ag-specificity could be inferred using TCR clustering algorithms (52, 53), or synthesized and expressed in cells to characterize experimentally (38).

Collectively, these data validate TAR-seq, a two-step method that identifies antigen-specific TCRs via highly specific TCR-induced gene expression signals and then utilizes those TCR sequences as barcodes to comprehensively identify antigen-specific T cells, irrespective of activation, and characterize their activity. This system provides specificity and precision relative to methods that solely rely on activation-induced signals, especially signals prone to non-specific background. Application of TAR-seq can characterize T cell activity *in vivo* with enhanced specificity and rigor, potentially identifying novel features of both efficacious and failed T cell immunity, which can be used to improve the design of vaccines and immunotherapies for infectious disease, cancer, and autoimmunity.

## Methods

### Animal subjects

All study macaques were housed at the Oregon National Primate Research Center (ONPRC) in animal biosafety level 2 rooms with autonomously controlled temperature, humidity, and lighting. Macaques were fed commercially prepared primate chow twice daily and received supplemental fresh fruit or vegetables daily. Fresh, potable water was provided via automatic water systems. During all protocol time points, body weight and complete blood counts were collected, and animals underwent anesthesia support and monitoring. The ONPRC Institutional Animal Care and Use Committee approved macaque care and all experimental protocols and procedures. Ketamine HCl (Ketathesia, Henry Schein Animal Health) with or without dexmedetomidine (Dexmedesed, Dechra) was used to sedate macaques for procedures. At the study end-point, macaques were euthanized with sodium pentobarbital overdose (>50 mg kg−1) and exsanguinated via the distal aorta. A board-certified veterinary pathologist performed and collected tissues at necropsy. The ONPRC is a Category I facility. The American Association for Accreditation of Laboratory Animal Care fully accredits the Laboratory Animal Care and Use Program at the ONPRC. It has an approved assurance (no. A3304-01) for the care and use of animals on file with the National Institutes of Health Office for Protection from Research Risks. The Institutional Animal Care and Use Committee adheres to national guidelines established in the Animal Welfare Act (7 U.S. Code, sections 2131–2159) and the Guide for the Care and Use of Laboratory Animals, Eighth Edition, as mandated by the U.S. Public Health Service Policy.

### Human subjects

This study was conducted according to the principles expressed in the Declaration of Helsinki. All ethical regulations relevant to human research participants were followed. The studies involving human participants were reviewed and approved by the Health Research Ethics Committee at Stellenbosch University (IRB0005240). Written informed consent to participate in this study was provided by the participants.

### Tissue collection and processing

Cell isolation from PBMCs and solid tissues were acquired and processed to single-cell suspensions using previously published methods, summarized below (5, 19). Liver, spleen, and mesenteric lymph node biopsies were collected by a minimally invasive laparoscopic procedure (39). For lung samples, animals were humanely euthanized, and caudal lung lobe samples were collected during necropsy. Bone marrow cells were harvested from the humerus or iliac crest by flushing with R10 media. PBMCs were isolated from freshly collected ACD-A treated blood utilizing Ficoll-Paque density centrifugation (GE Healthcare). Lymph nodes (LN) and spleen samples were homogenized as previously described (40) while liver and lung samples were enzymatically digested with DNAse and collagenase (41, 42). Prior to processing, cells were filtered using 70 um strainers. Cells were quantified using a Countess II (Thermo Fisher), aliquoted, diluted as required for single-cell RNA sequencing (typically 500-1,500 cells/uL), and kept on ice prior to processing. Mononuclear cells were isolated from bronchoalveolar lavage (BAL) as previously described (47).

### Vaccination and SIV challenge

The SIV-vaccinated RMs used for Gag CM9 analyses were vaccinated using a previously published approach (5). Animals were primed with vesicular stomatitis virus (rVSV) encoding full-length SIV Gag, boosted with SIV-Gag recombinant vaccinia virus (rVV) 8–9 weeks later, boosted with rVSV SIV-Gag 18 weeks later, and finally boosted with human adenovirus serotype 5 encoding SIV-Gag 12 weeks later. The RhCMV/SIV vaccinated macaques were each vaccinated with RhCMV 68-1/SIV vectors as previously described (54–56). RM5–8 were vaccinated with RhCMV 68-1/SIV vectors lacking viral protein PP71 (57), and RM9 received intact RhCMV 68-1/SIV vectors. This cohort was subsequently challenged intravenously with 5000 FFU SIVmac239M.

### MHC tetramer staining

Tetramer staining was performed similar to prior publications (38). PBMCs from RhCMV-infected Mamu-A1*002:01 (Mamu-A*02) RMs were isolated from anticoagulant-treated whole blood by Ficoll density gradient centrifugation (GE Healthcare). Tetramers for the Mamu-A*02-restricted RhCMV IE-2_313–322_ AN10, RhCMV IE-1_291–299_ VY9 and Mamu-A*01-restricted SIV Gag_181–189_ CM9 were provided by the NIH Tetramer Core Facility. For tetramer staining, approximately 1-2x10 (6) cells were placed in 100 μl of RPMI 1640 (with 10% FBS). Tetramers were added at a final concentration of 100nM and cells were incubated in the dark at 37 °C for 30 minutes. Subsequently, cells were stained for anti-CD3 (clone: SP34-2, Pacific Blue, BD Biosciences), anti-CD8 (clone: SK1, TruRed, BD Biosciences), anti-CD4 (clone: L200, PE-Cy7, BD Biosciences), anti-CD14 (clone M5E2, FITC, BioLegend), anti-CD16 (clone 3G8, FITC, BioLegend), and LIVE/DEAD Fixable Near Infra-Red Dead Cell Stain (Life Technologies) and were incubated for an additional 30 minutes in the dark at 4 °C. Samples were sorted by FACS and processed for 10x Genomics scRNA-seq as described below.

### Peptide stimulation and surface-trapped TNF-α staining assays

Antigen-specific CD8+ T cells were identified and viable cells were sorted using either surface CD69 or surface-trapped TNF-α staining using TAPI-0 (39). PBMCs, isolated CD8+ T cells (CD8+ T cell Isolation Kit, non-human primate, Miltenyi Biotec, 130-092-143), or isolated CD4+ T cells (CD4 MicroBeads, non-human primate, Miltenyi Biotec, 130-091-102) were incubated with peptide-pulsed antigen-presenting cells (K562, ATCC), co-stimulatory molecules CD28 (CD28.2, BD Biosciences) and CD49d (9F10, eBiosciences), anti-TNF (clone: MAb11, PE, BD Biosciences) and TAPI-0 (5nM final concentration, Santa Cruz Biotechnology). Cells were incubated at 37 °C for 8 hours unless otherwise indicated. After incubation, cells were stained with anti-CD3 (clone: SP34-2, Pacific Blue, BD Biosciences), anti-CD8 (clone: SK1, TruRed, BD Biosciences), anti-CD4 (clone: L200, PE-Cy7, BD Biosciences), anti-CD14 (clone: M5E2, APC, Biolegend), anti-CD69 (FN50, FITC, BioLegend), anti-CD16 (clone: 3G8, APC, Biolegend), anti-CD20 (clone: 2H7, APC, Biolegend) and LIVE/DEAD Fixable Near Infra-Red Dead Cell Stain (Life Technologies). Antigen-specific cells were defined as TNF+/CD69+ CD8+ T cells with responses 2x the magnitude of the no peptide control, with the no peptide control responses below 0.5%. Viable antigen-specific cells were sorted using a FACSAria Fusion (BD Biosciences), and analysis was conducted with FlowJo software (Tree Star). In addition to TNF+/CD69+ cells, CD69+ CD8+ T cells were also sorted. Cell stimulated with SEB (Toxin Technology Inc, BT202) were used as controls.

### SIV-infected cell recognition assays

For SIV-infected cell recognition assays, SIV-infected versus mock-infected autologous CD4+ T cells were used as stimulating antigens in conjunction with co-stimulatory anti-CD28 (CD28.2, Purified 500 ng/test: eBioscience, Custom Bulk 7014-0289-M050) and anti-CD49d mAb (9F10, Purified 500 ng/test: eBioscience, Custom Bulk 7014- 0499-M050) then incubated for 9 h presence of Brefeldin A (5 μg/ml; Sigma-Aldrich). Bead purified, negatively selected CD8+ T cells (CD8+ T cell Isolation Kit, non-human primate, Miltenyi Biotec, 130-092-143), derived from thawed PBMCs, were combined with autologous CD4+ T cells that were productively infected with SIVmac239. To generate SIV-infected CD4+ T cells, thawed PBMCs were positively selected by bead purification (CD4 MicroBeads, non-human primate, Miltenyi Biotec, 130-091-102) as per manufacturer’s instructions. Isolated CD4+ T cells were resuspended in R15, RPMI supplemented with 15% newborn calf serum (Hyclone, SH30401.01) 2 mM L-Glutamine (Gibco, 25030081), 100 U/mL Penicillin–Streptomycin Solution (Gibco, 15140122), and 100U/mL of IL2 (Peprotech, 200-02) then subsequently activated using anti-CD28, anti-CD49d, anti-CD3 (SP34-2, BD Biosciences, 551916), and SEB (Toxin Technology Inc, BT202) in 24-well plates, 2–6 million cells per well, and incubated at 37 °C. After 24 h, plates were washed 3 times with R15 then incubated for 3 additional days in R15 and 100U/mL of IL-2 with media changed as needed. Later, cells were infected with SIVmac239 by spinoculation and then incubated at 37 °C for 24H. SIV-infected cells were then combined with autologous negatively selected CD8+ T cells at an effector to target (E:T) ratio of 40:1 prior to standard flow cytometric ICS (plus or minus blocking reagents). Mock-infected autologous CD4+ T cells served as negative controls.

### Cell hashing

Cell hashing was used for most scRNA-seq samples, as previously described. Cells were labeled with the MULTI-Seq lipid labeling system (31), using commercially available lipid modified oligos (Sigma Aldridge LMO001). Cells were labeled with barcoded lipids as follows: 15uL MultiSeq solution 1 (LMO stock, diluted in PBS to 400nM) was added to the cell suspension, along with 45 uL of the barcode solution (10uM barcode oligo, diluted in PBS to 400nM), giving a final working concentration of 200nM for both LMO and the barcode oligo. The cell suspension was pipet mixed and incubated for 5 min at 4 °C. Next, 10uL of the MultiSeq co-anchor solution (50uM Co-A stock, diluted in PBS to 2uM) was added, followed by pipet mixing and incubation for 5 min at 4 °C. The cell suspension was washed twice with 1 mL cold PBS, spinning cells at 700 g for 5 min at 4 °C, and then resuspended in 200 uL R10 to quench LMOs. Samples were pooled, followed by GEM generation on the 10x instrument.

### *M. tuberculosis* auxotroph preparation

*M. tuberculosis* (*Mtb*) auxotroph mc26206 (H37Rv ΔpanCD ΔleuCD) (58) was as a kind gift from Bill Jacobs (MTA-IN19-168) and is modified such that it is only able to grow in the presence of externally supplemented pantothenate and leucine and thus can be handled in a Biosafety Level 2 setting. The *Mtb* auxotroph was grown to OD600 0.5 in 7H9 broth supplemented with Leucine (50μg/ml), Pantothenate (24μg/ml), and 5% Tween 80.

### Dendritic cell isolation, *Mtb*-infection, and co-culture assays

Human monocyte-derived dendritic cells (DCs) were isolated from Peripheral Blood Mononuclear Cells (PBMCs) by plate adherence. For this, PBMCs were incubated in RPMI supplemented with 2% heat-inactivated human serum, 3.5 mM L-Glutamine (Gibco, 25030-081), and 44 μg/ml gentamicin (Gibco, 15750-060) at 37 °C for 1h, before gentle tapping and removal of non-adherent cells. Adherent cells were then cultured in RPMI (Gibco, 2240-089) supplemented with 10% heat-inactivated human serum (RPMI10%HuS) and 30 ng/ml GM-CSF (Leukine, Partner Therapeutics) and 10 ng/ml IL-4 (R&D Systems, 204-IL-001MG/CF) for 5 days (59). Per sample, 1e5 DCs were either uninfected or infected with *Mtb* auxotroph at a multiplicity of infection (MOI) of 9:1 and incubated overnight.

Separately, PBMC were thawed in the presence of DNase, resuspended in RPMI10%HuS and CD3+ T cells were isolated using negative selection with the Pan T cell Isolation Kit (Miltenyi Biotec, 130-096-535) per the manufacturer’s instructions. *Mtb*-infected and uninfected DCs (1e4) were then incubated with T cells (1e5) overnight. Cells were then processed for 10x Genomics single cell RNA-seq, as described below.

### Single-cell RNA sequencing

Single-cell RNA sequencing was performed using the 10x Genomics platform, as previously described. The isolated single cell suspensions were processed for single-cell RNA sequencing using the 10x Genomics Chromium platform, using 5’ v2 or HT chemistry, following the manufacturer’s protocols, including feature barcoding library preparation. To improve capture of MULTI-Seq fragments, we added the following primer, 5’-CCTTGGCACCCGAGAATTCC-3’, at 2.5uM to the 10x cDNA synthesis step. Generation of VDJ enriched libraries followed manufacturer’s instructions with the exception that macaque-specific TCR constant region primers were used in place of human-specific TCR enrichment primers for macaque cells (47). Primer pairs were used to amplify the alpha, beta, delta, and gamma TCR chains. The concentration of the alpha constant region primer was increased relative to the beta primer to improve amplification. PCR conditions for both reactions were as follows: lid temp 105 °C, 98 °C 0:45, 12 cycles of: 98 °C for 0:20s, 60 °C for 0:30s, 72 °C for 1:00 min, followed by 72 °C for 1:00 min and 4 °C hold. Sequence libraries were sequenced using Illumina chemistry, on either Novaseq or HiSeq instruments (Illumina).

### Single-cell RNA-seq processing

Analysis of scRNA-seq was conducted, largely as previously described. Raw sequence reads were processed using 10X Genomics Cell Ranger software (version 8.0.1). The resulting sequence data were aligned to the MMul_10 genome (assembly ID: GCF_003339765.1) with NCBI gene build 103. Cell demultiplexing used a combination of algorithms, including GMM-demux, demuxEM and BFF, implemented using the cellhashR package (60–62). Droplets identified as doublets (i.e. the collision of distinct sample barcodes) were removed from downstream analyses. We additionally performed doublet detection using DoubletFinder and removed doublets from downstream analysis (63). Data were otherwise processed as previously described (47). Analyses utilized the Seurat R package, version 5.4 (64). A complete listing of the SRA accession numbers for datasets generated for this manuscript are available in **Supplementary Table 3**, with processed gene count data submitted to the NIH Gene Expression Omnibus database under accession GSE320096 and NIH BioProject PRJNA1425055. The Rhesus Macaque Immune Atlas (RIRA) dataset was used for multiple analyses, with the input gene expression data from NIH GEO database, accession GSE277821, and NIH BioProject PRJNA1163395 (47).

### Training dataset assembly and annotation

We developed a supervised learning framework using a multi-class training dataset comprising single-cell RNA sequencing (scRNA-seq) profiles from TCR-stimulated CD4+ and CD8+ T cells (with and without BFA), bystander T cells (with and without BFA), unstimulated cultured T cells (with and without BFA), and bulk uncultured T cells isolated from multiple tissues (No BFA). TCR-stimulated, unstimulated cultured, and bystander T cells were obtained from previously described antigen-stimulation experiments. Bystander T cells were defined as those exposed to identical culture and antigen conditions but lacking antigen-specific clones, as determined by tetramer staining and transcriptional profiling, as previously described.

Bulk tissue T cells were sourced from the Rhesus Macaque Immune Atlas (RIRA) dataset and defined as cells with an effector differentiation score (EDS) greater than 6. These cells served as negative controls, representing tissue-resident, non-TCR-stimulated states, and were included to prevent conflation of tissue residency or homing programs with stimulation-induced transcriptional signatures.

Within the stimulated compartment, TCR-stimulated CD4+ and CD8+ T cells were further annotated into transcriptionally distinct functional states based on differential gene expression and unsupervised clustering. Th1-like cells were characterized by high expression of cytotoxicity- and cytokine-associated genes, including *IFN-γ*, *TNF-α*, *GZMB*, and *CCL3*. Th2/Th17-like cells were identified by elevated expression of lineage-associated genes, such as *CCR4*, *TSHZ2*, *AFF3*, *PELI2*, *GPHN*, and *LDLRAD4*, consistent with previously described Th2 and Th17 transcriptional programs (43). Th1-like cells were further subdivided into three groups according to MIP-1β and CD137 expression: MIP-1β+, MIP-1β- CD137+, and MIP-1β- CD137-. Unstimulated cultured T cells formed two distinct clusters, with BFA treatment as the main axis of separation. Group identity for these cells was based on cluster membership rather than treatment annotation; cells co-clustering with the opposite treatment mostly inherited the corresponding cluster label. As a result, 96% of unstimulated cultured with BFA T cells and 97% of unstimulated cultured without BFA T cells assumed a label that matched their BFA treatment. These curated annotations were used as class labels for supervised model training.

### Supervised feature extraction using sPLS-DA

We applied Sparse Partial Least Squares Discriminant Analysis (sPLS-DA) using the mixOmics framework version 6.34 for supervised multivariate feature extraction, aiming to identify low-dimensional transcriptional components that maximally discriminate among cellular classes while enforcing gene-level sparsity. Prior to modeling, we filtered genes with near-zero-variance using mixOmic’s nearZeroVar function.

(1) $$\underset{u_{h},\;v_{h}}{\min}∥M_{h}-u_{h}v_{h}^{T}{∥}_{F}^{2}+P_{\lambda }(u_{h})\;$$

The sPLS-DA method in mixOmics is an iterative method, minimizing the Frobenius norm of Equation 1. Applied to our dataset, $M=X^{T}Y$ where M is the predictor-class cross-covariance matrix, $X^{T}$ is the matrix of scaled and normalized gene expression data, and Y is indicator matrix of cellular classes, which together are deflated to $M_{h}=X_{h}^{T}Y_{h}$ over the h components. We defined h=9 based on hyperparameter tuning, described below. The vectors $u_{h}$ and $v_{h}$ are iteratively weighted left (gene-wise) and right (class-wise) weight vectors of the same h dimensions. The $P_{\lambda }(u_{h})$ term is a shrinkage operator, inducing sparsity into the gene-wise weight vector $u_{h}$. MixOmics does not expose the regularization parameter λ directly but enables users to set parameters for a desired number of genes and implicitly derives an approximately equal λ, which we estimated via hyperparameter tuning.

Hyperparameter tuning was performed on a balanced, randomly sampled subset of the data with 500 cells per class via 5-fold cross-validation, repeated 10 times. The number of components (ncomp parameter) spanned 1 to 10, with 9 being the optimal number of components. Candidate sparsity levels for each component (keepX parameter) were evaluated over variably spaced grids spanning 20 to 100 in increments of 10 and 120 to 320 genes in increments of 40. The optimal sparsity profiles for each component were: 240, 280, 320, 80, 30, 20, 240, 120, and 320 for components 1 through 9, respectively. Model performance during tuning was assessed using the balanced error rate (BER) as the optimization criterion, with class discrimination based on the Mahalanobis distance. All parameters were then treated as fixed and used to train the final sPLS-DA model on the complete dataset.

### Probabilistic classification using multinomial logistic regression

Although the mixOmics framework provides class prediction by projecting new samples into the sPLS-DA component space, class centroids are computed during the prediction step which is computationally infeasible for the finalized model trained on 222,350 cells. To address this, we used the cellular scores (variates) from the sPLS-DA model to train a multinomial logistic regression model using nnet’s multinom method (46). The cellular class labels were used as the response variable. Cells were randomly split into training and test sets at a 50/50 ratio. The multinomial logistic regression model was then fit on the training subset using component scores as predictors and multi-class cell-state labels as outcomes. Model parameters were estimated by maximum likelihood, with class membership probabilities parameterized through the softmax function (Equation 2) where i is the sPLS-DA’s component index, j is a specific cellular class, k the iterator over the cellular classes, and $X_{i}$ are the variates ( $u_{h}X$, as defined in the previous section). We set the non-specific bystander T cells treated with BFA to be the reference class and use the reference class parameterization for the softmax accordingly.

(2) $$P(Y=j)=\frac{e^{\beta_{ij}X_{i}}}{1+\sum_{\left\{\text{k}=1\right\}}^{\text{K}-1}e^{\beta_{k}X_{i}}}$$

The trained classifier was subsequently applied to the held-out test set to generate predicted class labels and class membership probabilities. Model performance was evaluated using a confusion matrix comparing predicted and true class labels (**Figures 3C, E–G**). To facilitate interpretation across classes with differing abundances, confusion matrices were row-normalized so that each row represented the proportion of cells from a given true class assigned to each predicted class.

### Definition of the unified TCR stimulation probability score

To define a continuous measure of TCR stimulation applicable across TCR-stimulation states and T cell lineages, we defined a unified TCR stimulation probability score based on the output of the multinomial logistic regression classifier. For each cell, the classifier generates a vector of probabilities reflecting membership in each annotated cell-state class. We sum these probabilities, specifically across the MIP-1β+, MIP-1β- CD137+, MIP-1β- CD137-, and Th2/Th17-like classes in Equation 2 to define the unified TCR stimulation score.

### Threshold selection for gating of antigen-responsive cells

For threshold definition, cells in the reference CD8+ dataset were first binarized into two groups: Antigen-specific/TCR-stimulated cells and Other, which included all non–antigen-specific, bystander, and unstimulated populations. Thresholds for the unified TCR stimulation probability score were defined using both probabilistic interpretation and the distributional properties of the negative class.

A lenient (permissive) threshold of 0.1 was set as the threshold to capture lower-confidence antigen-responsive cells. This value lies within the upper tail (98^th^ percentile of CD8+ T cells, 99^th^ percentile of CD4+ T cells) of the stimulation score distribution in the negative class. We assessed the performance of this threshold based on ROC curves and corresponding AUCs computed by the pROC R package (65). In addition to the AUCROCs, we computed nonparametric Cliff’s delta and pairwise unpaired Wilcoxon rank sum tests with a Holm-Bonferroni adjustment. We did not formally assess that the *a priori* majority-probability threshold (0.5) had favorable classification characteristics, but infer it is more stringent via the Neyman-Pearson lemma (66).

### Gene modules used for T cell differentiation and function

T cell differentiation and functional programs were measured using gene modules and the UCell R package (48). Cytotoxic differentiation was measured using granzyme (*GZMA*, *GZMB*, *GZMH*). Highly cytotoxic T cells were identified using a single gene UCell based on *PRF1*. To mitigate gene dropout, this score was smoothed using the UCell SmoothKNN function to compute weighted averages of the k nearest neighbors with the default parameters (k = 10), with a threshold of 0.8. Cytokine expression was measured using a UCell module based on *TNFRSF14*, *CCL3*, *CCL4L1*, *NFKBID*, *IFNG*, and *TNF* (threshold of 0.5). Cells were defined as dividing using the following: 1) unsupervised clustering was performed as described above, 2) clusters were inspected for expression of *MKI67* and *TOP2A*, 3) all cells belonging to the cluster enriched for these genes are assigned as dividing.

### Gene module scoring and pathway analysis

TCR stimulation gene modules were derived from differential expression analysis of CD8+ T cells (**Figure 1**; **Supplementary Figure 4**). The top markers for clusters 3 and 5 were selected as candidates for module scoring. Module scores were computed for each cell using the UCell R package. The performance of each module was assessed using ROC analysis, and AUC values were computed with the pROC R package. Pathway analyses were performed using enrichGO in clusterProfiler (67) under Benjamini-Hochberg multiple hypothesis correction.

### Mapping between TCR libraries and V/J data from cells

The TAR-seq ex vivo stimulation experiments identify a list of paired alpha/beta CDR3 sequences that are antigen specific in a subject. These TCRs can be matched against new scRNA/TCR-seq data from that subject, such as unsorted T cells, to identify the Ag-specific cells. The 10x genomics detection of TCR sequence is not perfect and can result in cells with missing data (i.e., lacking data for either the alpha or beta chain). To address this, cells are categorized as Ag-specific using the following logic: 1) if that cell has data for both alpha and beta chains, then it can be categorized as Ag-specific only when both chains match clonotype from the TCR library, 2) if that cell has data for one chain and no data for the other, this is assumed to be a technical artifact and assignment is based on only one chain, 3) if the cell has data for both chains and one matches but the other does not, it is not categorized as Ag-specific. The last case is highlighted in **Figure 5** (dashed bars).

## Data Availability

The datasets presented in this study can be found in online repositories. The names of the repository/repositories and accession number(s) can be found in the article/**Supplementary Material**.

## Supplementary material

Supplementary Table 1 Datasets used to train a probabilistic model to identify TCR-stimulated T cells. Dataset composition for sPLS-DA training is shown as cell counts and proportions. Columns indicate: (i) original experimental condition (*ex vivo* or *in vivo*), (ii) labels used for model training, (iii) *post hoc* recoded labels mapped to conventional nomenclature, and (iv) consensus class labels that simplify these categories into T cell classes.

Supplementary Table 2 Model components and gene weights. Features contributing to each of the nine latent components of the trained sPLS-DA model and their corresponding weights. Weight magnitude reflects contribution strength to the component, and the sign indicates direction of association with the modeled class structure.

Supplementary Table 3 SRA accession numbers and metadata for single-cell RNA/TCR-seq datasets.
