# Supplementary material for: T cell antigenic recognition sequencing (TAR-seq) enables precise characterization of vaccine-elicited cellular immunity ex vivo and in vivo
Source: Front Immunol. 2026 Jul 22;17:1823042. doi: 10.3389/fimmu.2026.1823042 (PMC13438159; doi:10.3389/fimmu.2026.1823042)
Supplement: Supplementary file 1 [file Image1.pdf]

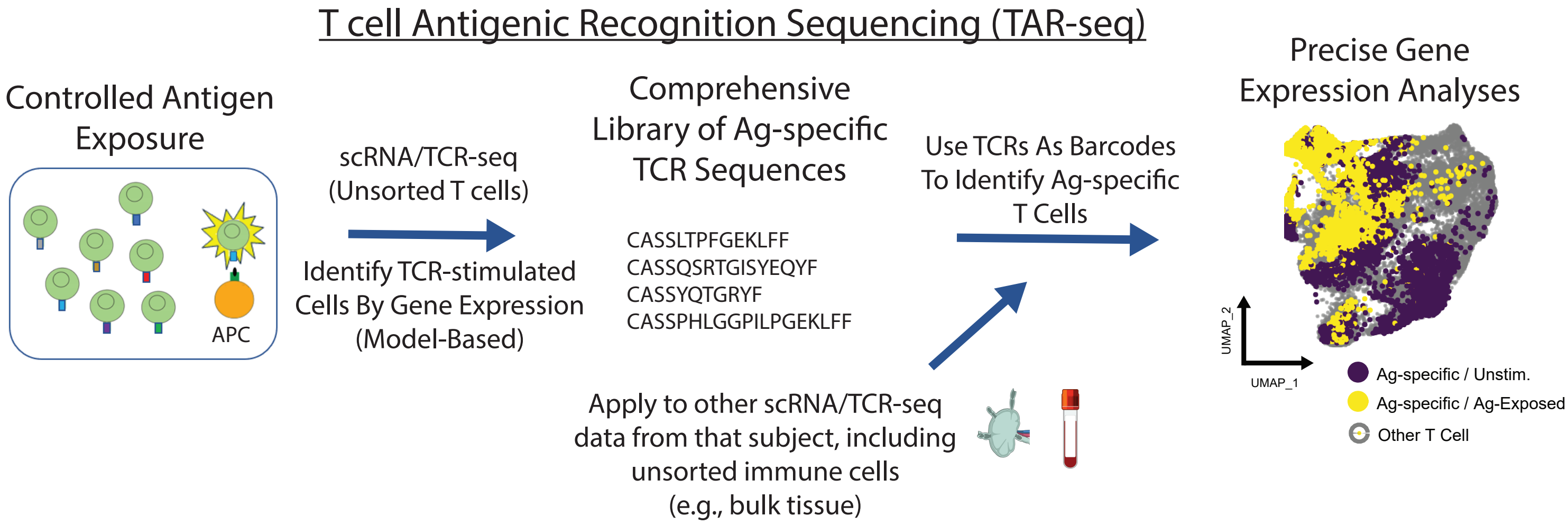
T cell Antigenic Recognition Sequencing (TAR-seq)
Controlled Antigen Exposure
APC
scRNA/TCR-seq (Unsorted T cells)
Identify TCR-stimulated Cells By Gene Expression (Model-Based)
Comprehensive Library of Ag-specific TCR Sequences
CASSLTPFGEKLFF
CASSQSRTGISYEQYF
CASSYQTGRYF
CASSPHLGGPILPGEKLFF
Use TCRs As Barcodes To Identify Ag-specific T Cells
Apply to other scRNA/TCR-seq data from that subject, including unsorted immune cells (e.g., bulk tissue)
Precise Gene Expression Analyses
UMAP_2
UMAP_1
Ag-specific / Unstim.
Ag-specific / Ag-Exposed
Other T Cell
