# Supplementary material for: T cell antigenic recognition sequencing (TAR-seq) enables precise characterization of vaccine-elicited cellular immunity ex vivo and in vivo
Source: Front Immunol. 2026 Jul 22;17:1823042. doi: 10.3389/fimmu.2026.1823042 (PMC13438159; doi:10.3389/fimmu.2026.1823042)
Supplement: Supplementary file 2 [file DataSheet1.docx]

**Supplemental Figures**

**Figure S1. Identification and validation of CD4+ T cell gene signatures specific to TCR stimulation.** All panels display single-cell RNA/TCR-seq (scRNA/TCR -seq) data from two RMs, either stimulated with SEB or unstimulated, without brefeldin A (BFA). **A)** A UMAP projection of 36,026 CD4+ T cells. **B)** The bar plot summarizes the proportion of cells for each cluster in **(A)** derived from the SEB or No Stim conditions. **C)** Dot plot of differentially expressed genes between clusters in **(A)**. **D, E)** Temporal stacked bar plot showing time point proportions per cluster, stratified by BFA treatment.

**Figure S2. TRBV gene frequencies in clusters activated after SEB stimulation of CD4+ T cells.** The panel summarizes the frequency of each TRBV gene in the SEB-enriched clusters relative to other cells.

**Figure S3. Kinetics of activation-induced gene expression in CD8 T cells.** All plots summarize the model training data shown in main Figure 3. **A)** Violin plots display the per cell sPLS-DA component scores for each component within the training data. **B)** The plots display the top positively and negatively weighted genes for the indicated components, demonstrating that the model heavily weights many canonical activation-associated markers. **C)** The plot displays the top pathways enriched for the indicated components. **D)** CD8 T cells were stimulated with antigenic peptide-pulsed antigen-presenting cells or mock-stimulated. Cells were incubated for 2-24 hours, followed by scRNA/TCR-seq. Each plot displays the average expression of the indicated marker gene, at the indicated time point. Expression is calculated separately for antigen-specific (Ag-specific) and other T cells.

**Figure S4. Comparison of probabilistic TCR stimulation model and simple gene modules.** All panels use the same model test data as Figure 3. **A)** Heatmap illustrating gene membership across six UCell scoring modules. The displayed genes represent the top differentially expressed genes co-expressed in clusters 3 and 5 from the CD8+ T cell analysis (Figure 1), including canonical markers of TCR activation and T cell stimulation. Blue tiles indicate the inclusion of each gene within the respective module. Module scores were computed using the UCell R package. AUROC values for classifying TCR-stimulated cells are shown for both CD4+ (orange, top) and CD8+ (orange, bottom) T cells. Module 6 exhibits the highest performance in both subsets (AUROC = 0.95 and 0.99, respectively). **B)** ROC curves compare the TAR-seq TCR stimulation score (orange) with the best-performing UCell module (Module 6, blue) for CD8+ (left) and CD4+ (right) T cells. TAR-seq achieved AUC values of 0.999 and 0.992 in CD8+ and CD4+ T cells, respectively, compared to 0.993 and 0.946 for Module 6. **C)** Sensitivity of TCR-stimulated cell detection at background-matched score thresholds (1% false positive rate among non-stimulated background cells) for CD8+ (left) and CD4+ (right) T cells. Bar heights indicate the mean proportion ± SE of antigen-specific/TCR-stimulated (left bars) and background (right bars) cells exceeding the threshold for the TAR-seq TCR stimulation score (orange) and the best performing UCell module (blue).

**Figure S5. Applicability of TCR stimulation model to human T cells.** All panels summarize data from CD3+ T cells isolated from PBMCs of three TB exposed donors. T cells were incubated with autologous DCs that were either Mtb-infected or mock-infected. **A)** UMAP embedding showing unsupervised clustering of T cells co-cultured with either TB-infected or uninfected dendritic cells (DCs). Five clusters enriched for TB-infected DC stimulation are designated TB-1 to TB-5, with remaining clusters grouped as "Other." **B)** Stacked bar chart shows the proportional composition of each cluster by stimulation condition (TB-infected DCs, orange; uninfected DCs, blue), illustrating differential enrichment of TB-infected DC-stimulated cells across clusters. **C–D)** UMAP feature plots show normalized expression of IFN-γ and TNF-α, respectively. Expression is highest in clusters TB-1 and TB-5, consistent with antigen-specific stimulation. **E)** UMAP visualization of the TAR-seq TCR stimulation score shows high scores concentrated in TB-1 and TB-5. **F)** Binary TAR-seq classification of TCR-stimulated cells (blue) confirms localization of predicted stimulated cells to the same clusters. **G)** Dot plot of differentially expressed genes across clusters, grouped by cluster identity. Clusters TB-1 and TB-5 are enriched for TCR activation and effector markers. Clusters not enriched for TCR stimulation associated genes (TB-2, TB-3, TB-4) show enrichment for lineage-associated markers, including cytotoxic T cell signatures. Dot size reflects the proportion of cells expressing each gene; color indicates scaled mean expression. **H)** Per-donor quantification of antigen-specific clonotypes identified using TAR-seq stimulation predictions. Bar height represents the fraction of total T cells classified as TCR-stimulated for each donor under uninfected or TB-infected DC conditions. Each bar is subdivided by clonotype, where colored fills represent individual antigen-specific clones and gray fills represent low-frequency clones. **I)** UMAP highlights the distribution of antigen-specific cells by stimulation condition, depicting cells with TB-reactive TCRs co-cultured with TB-infected DCs (purple) and those with uninfected DCs (yellow). Antigen-specific cells are enriched in clusters TB-1, TB-2, and TB-5, indicating the main transcriptional states assumed by TB-reactive T cells under different stimulation conditions.

**Figure S6. Basal rates of CD8 T cell activation signatures in healthy RMs.** All panels summarize scRNA-seq data from the RIRA cell atlas, a public dataset comprised of multiple tissues from healthy control RMs. **A)** RIRA cells were scored using the probabilistic TCR stimulation model. The graph displays the percentage of cells classified as activated. **B)** The graph displays the percentage of healthy control CD4+ and CD8+ T cells expressing proliferation markers. **C)** The graph displays the percentage of healthy control CD4+ and CD8+ T cells enriched for a cytokine gene module. **D)** The graph displays the percentage of healthy control CD4+ and CD8+ T cells enriched for a cytotoxicity gene module, based on perforin expression. These data demonstrate that most measurements of T cell activity have low but non-zero basal rates in healthy RMs.
